# Post-traumatic stress disorder among military personnel admitted at the Northwest Command Level Three Military Hospital, Bahir Dar, Ethiopia, 2022: an institution-based cross-sectional study

**DOI:** 10.3389/fpsyt.2024.1410630

**Published:** 2024-09-18

**Authors:** Assasahegn Tedla, Sintayehu Asnakew, Getasew Legas, Birhanu Mengist Munie, Minale Tareke, Micheal Beka

**Affiliations:** ^1^ Department of Psychiatry, School of Medicine, College of Health Science, Debre Tabor University, Debre Tabor, Ethiopia; ^2^ Department of Psychiatry, School of Medicine, College of Health Science, Bahir Dar University, Bahir Dar, Ethiopia

**Keywords:** depression, Ethiopia, military personnel, PTSD, prevalence

## Abstract

**Background:**

Post-traumatic stress disorder (PTSD) is one of the most common mental health problems that military personnel encounter. It could be lifelong and affect every aspect of military personnel, including their mental and physical health, family and social interactions, and their work. However, in Ethiopia, the magnitude and its associated factors have not been well investigated.

**Objective:**

This study aimed to determine the prevalence of PTSD and its associated factors among military personnel, who were admitted at the Northwest Command Level Three Military Hospital, Bahir Dar, Northwest, Ethiopia, 2022.

**Methods:**

An institution-based cross-sectional study was conducted from 21 June to 21 July 2022, at the Northwest Command Level Three Military Hospital. A computer-generated simple random sampling technique was used to select a total of 627 participants. The 17-item Military Version Checklist was utilized to measure PTSD. The Patient Health Questionnaire, Brief Resilience Coping, and Critical War Zone Experience scale were utilized to measure depression, resilience, and combat exposure, respectively. Descriptive, bivariate, and multivariate binary logistic regressions with odds ratios and a 95% confidence interval were used. The level of significance of the association was determined at a *p*-value < 0.05.

**Results:**

A total of 612 respondents participated, with a response rate of 97.6%. The prevalence of PTSD in this study was 21.9% (95% CI: 18.6, 25.2). In multivariable regression, female sex [adjusted odds ratio (AOR) = 2.3, 95% CI; 1.3, 3.87], combat personnel (AOR = 2.75, 95% CI; 1.44, 6.36), handling dead bodies (AOR = 2.5, 95% CI,1.24, 5.02), having 4–5 deployments (AOR = 2.94, 95% CI, 1.63, 5.32), having ≥6 deployments (AOR = 3.4, 95% CI, 1.95, 6.17), low resilience coping (AOR = 2.02, 95% CI; 1.16, 3.53), poor social support (AOR = 2.46, 95% CI, 1.39, 4.35), very high combat exposures (AOR = 4.8, 95% CI, 2.03, 11.93), and depression (AOR = 2.8, 95% CI, 1.68, 4.67) were significantly associated with PTSD.

**Conclusion:**

PTSD is markedly prevalent among the Ethiopian military population, with key risk factors identified as being female, poor social support, low resilience coping skills, handling dead bodies, multiple deployments (four or more), high combat experiences, and depression. Healthcare professionals must prioritize the early diagnosis and intervention of PTSD in vulnerable groups of military personnel.

## Introduction

Post-traumatic stress disorder (PTSD) is a syndrome that arises from exposure to actual or potential death, sexual assault, and military combat (1). PTSD has a significant impact on public health, with approximately 8 million individuals developing PTSD each year (2), contributing to 0.4% of years lived with disability (3), and approximately 3 million disability-adjusted life years are associated with PTSD in low- to middle-income countries (LMICs) (4).

PTSD is a common mental disorder, with slightly higher rates among military personnel (13%) compared to the general population (6%) (5). This might be because military personnel experience more combat trauma and paramilitary trauma than civilians (6). Hence, exposure to trauma is a prerequisite for PTSD (7, 8). Additionally, military personnel experience various stressful situations, including repeated exposure to death and injuries, exhaustion and deprivation, and separation from family and friends (9–11). Furthermore, approximately 44%–72% of veterans experience high levels of stress after returning to civilian life (12) and they are at high risk of violence and aggressive traumatic-related disorders (13). Together, these conditions contribute to military personnel being more prone to PTSD.

Approximately 50% of those who have been in the military have suffered mental health problems, including PTSD (14). Similarly, studies revealed that one in five (22%) of the Australian defense force population had experienced a mental disorder in the previous 12 months (15).

PTSD is one of the signature injuries; an estimated 13%–20% of more than 2.6 million troops deployed in Iraq and Afghanistan suffer from PTSD (16). PTSD is the most frequent mental illness among military people, with a lifetime prevalence rate of 22% (17). Notably, it affects 16.8% of Australian veterans (18), 7.6% of Canadian armed forces members (19), 29% of China’s veterans (20), and 25.8% of individuals serving in the South African defense force (6). Furthermore, the prevalence ranges from 6% to 31% among combat veterans in the United States (21).

PTSD in the military causes increased suicidal behavior (22), mounting substance use behaviors (23), difficulties in the mental health of spouses (24), reduced quality of life, impaired work performance, and a decline in the quality of family life (25). PTSD is frequently associated with other mental illnesses; 83.3% of PTSD patients had a co-occurring mental illness (26). Since then, active military personnel with comorbid psychological conditions had more severe symptomatology, lower rates of recovery, higher attrition from service, and higher rates of attempted and completed suicide (27).

Despite the significant burden of PTSD among the military population, over 80% of armed forces members with mental illness did not receive medication or counseling (28). Consequently, untreated mental health conditions can lead to a decline in operational readiness, an elevated likelihood of premature separation from the military, and mortality due to suicide (29). Especially in Ethiopia, like many LMICs, primary care clinics are staffed by providers with limited training in mental health, and high staff turnover (30). This might be contributing to Ethiopia’s mounting burden of providing mental health services to military personnel.

The study in Ethiopia is limited; in a previous study, the prevalence of PTSD among Ethiopian military personnel was approximately 16% (31). Additionally, some factors such as time spent in forwarding, coping, and resilience, which were significantly associated in most others studies, were added in this study. Thus, this study aimed to determine the prevalence of PTSD and its associated factors among military personnel in a military treatment center at the Northwest Command Level Three Military Hospital in Bahir Dar.

## Materials and methods

### Study area and study period

The study was conducted from 21 June to 21 July 2022, at the Northwest Command Level Three Military Hospital, located in Bahir Dar city, in the Amhara region, approximately 565 km northwest of Addis Ababa, the capital city of Ethiopia. Since the rise of the conflict in northern Ethiopia between the Tigray People’s Liberation Front party and the Ethiopian National Defense Forces (32), the attacked and injured soldiers have been receiving treatment at the Northwest Command Level Three Military Hospital, which is the Ethiopian Defense Force North West Command’s residential area near Bahir Dar.

Since various hospitals provide treatments for injured troops, including the Northwest Command Level Three Military Hospital, the hospital serves as the base hospital for casualties. It renders dental, psychiatric, medical, and surgical services to outpatients and medical and surgical services to inpatients. Two Bachelor of Science (BSc) psychiatry professionals provide outpatient psychiatric services.

### Study design and population

An institution-based cross-sectional study design was conducted. All admitted military personnel at the Northwest Command Level Three Military Hospital are the source population, whereas all admitted military personnel who were randomly selected during the data collection period were the study population.

### Inclusion and exclusion criteria

All admitted military personnel during the data collection period who were capable of responding to the questionnaire were included in the study, whereas individuals who were seriously ill (failure to respond due to severe pain and unable to communicate) were excluded.

### Sample size determination

The sample size was determined by using a single population proportion formula based on the estimated prevalence rate of PTSD of 16% taken from the previous research conducted in Ethiopia (31), with a 95% confidence level and 3% margin of error to increase the sample size, while applying the formula and the final sample size of 627 with a 10% non-response rate.

### Sampling procedure

In this study, a simple random sampling technique was applied. To establish a sampling frame, we executed bed number labeling. To begin, a list of military personnel from all wards was compiled to create a single sampling frame. After selecting the military personnel and their bed numbers, assigned and labeled by four data collectors 2 days before the data collection period, a single sample frame was established. Finally, a computer-generated simple random number was used to pick study participants.

### Data collection procedures

The questionnaire was initially written in English and translated into the local Amharic language by language experts. The questionnaire was then translated back into English by an independent person to verify the consistency and comprehensibility of the tool. A pre-test was also conducted on 5% (32) of participants at the Feleg hiwot compressive specialized hospital (which rendered service to military personnel during the conflict) to ensure the clarity of the questionnaires. Four BSc generic nurse data collectors and two BSc psychiatry nurse supervisors were recruited. Before data collection, the data collectors provided a comprehensive explanation of the study’s objectives to every participant. Subsequently, the participants were requested to indicate their willingness and provide written consent. Finally, the participants filled out the questionnaires provided to them. Data quality was ensured by training the data collectors 3 days before data collection and continuous monitoring of the daily evaluation of each completed questionnaire by the principal investigator, who holds a Master of Sciences degree in mental health.

### Data collection tools

The military version of the PTSD questionnaire (PCL-M) was used. The PTSD Checklist-Military Version is a self-report rating scale that assesses the severity of PTSD symptoms in the military. Participants respond with a five-point Likert scale ranging from 1 (not at all) to 5 (extremely) (1 = not at all, 2 = a little bit, 3 = moderately, 4 = quite a bit, and 5 = extremely). This score is calculated by summing all of the scale item responses together. The overall score can vary from 17 to 85, with higher levels indicating more seriousness. Ratings are based on current DSM criteria, based on how much a veteran has been affected by a specific traumatic military-related event, with a sensitivity of 0.82, a specificity of 0.83, and an internal consistency of 0.97 (33). The local language-translated version of PCL-M has demonstrated good reliability in this study, exhibiting a Cronbach’s alpha coefficient of 0.93. The PCL-M has been employed in prior research conducted in Ethiopia, demonstrating commendable reliability in assessing PTSD among military personnel receiving hospital care (31). Furthermore, it has been utilized in numerous studies conducted on military populations (34, 35).

The Patient Health Questionnaire (PHQ-9), a nine-item self-report questionnaire, was employed to assess depression, with a four-point ordinal scale used for scoring purposes. Individuals with a PHQ-9 score equal to or exceeding 10 were screened for depression, with scores ranging from 5 to 27. Validation studies conducted in adult populations in Ethiopia indicated a sensitivity of 86% and a specificity of 67% for the PHQ-9 (36). PHQ-9 has good measure of depression among the military population.

Combat exposure was measured on the Critical War Zone Experience scale. The combat exposure scale consisted of seven items assessing experiences. Participants were asked to indicate how often they experienced each combat stressor using a five-point Likert scale from 0 (“never”) to 4 (“10+ times”) and to respond based on the frequency of their experiences, such as “seeing ill or injured women or children who you were unable to help,” “being directly responsible for the death of an enemy combatant,” and “having a buddy shot or hit who was near you.” It was validated for PTSD in veterans’ clinics. The scale has a high alpha coefficient of 0.73 (37). The overall combat exposure score was created by summing across all scale items. The level of combat exposure was classified into four groups (low, medium, high, and very high) in accordance with a similar study done previously (38).

The patient’s life threat was measured using the perceived stress scale, which has possible scores ranging from 0 to 40 on a five-point Likert scale (0 = never, 1 = almost never, 2 = sometimes, 3 = fairly often, and 4 = very often). This tool was validated in Ethiopian university students, and its Cronbach’s alpha value of internal consistency was 0.80 (39).

The Brief Resilience Coping Scale measures a five-point scale response ranging from 1 (does not describe me at all) to 5 (very well describes me). Total sum scores range from 4 to 20. Scores of 4–13 indicate low resilient coping, 14–16 indicate medium resilient coping, and 17–20 indicate high resilient coping, which have adequate internal consistency (*r* = 0.76) and test–retest reliability (*r* = 0.71) (40). The Oslo-3 scale, which ranges from 3 to 8, was used to assess social support. The total score ranged from 3 to 14 on the OSS-3 questionnaire; sub-domain scores of “3–8,” “9–11,” and “12–14” indicate poor social support, intermediate social support, and strong social support, respectively (41).

Types of combat exposures were assessed as yes or no and were adapted from the Army Mental Health Advisory Team combat exposure scale and different literature (38). The patients’ background information was assessed using a questionnaire that included sociodemographic and military-related demographic data. Child abuse (physical, sexual, and neglect) was assessed with yes or no questions. The respondents’ yes/no responses were analyzed to evaluate their history of substance use and clinical factors, which will be operationalized based on different literary works.

### Data processing and analysis

After ensuring the completeness of the data, it was entered into Epi Data version 4.46 and then exported to Statistical Package for Social Science version 25.0. Dependent and independent variables in the study were described using descriptive statistics such as mean, standard deviation, frequency, and percentage. The results were visually presented through graphs and tables.

To identify PTSD and related factors, bivariate and multivariate binary logistic regression analyses were used. Variables that are significant in bivariate analysis (with a *p*-value 0.25) were considered for multivariable logistic regression analysis. The adjusted odds ratio (AOR) at the 95% confidence interval was used to evaluate the strength of the association. A *p*-value of 0.05 in multivariate logistic regression was deemed statistically significant. The goodness of the model fit was evaluated using the Hosmer and Lemeshow test, yielding a value of 0.93.

## Results

A total of 627 participants with a response rate of 97.6% participated in this study. The mean age of the respondents was 30 years, with a standard deviation of ±6.4 years. Among the participants, 278 individuals (36.6%) were aged 30 years and older, with 466 (76.1%) being men and 261 (42.6%) identifying as orthodox followers. As regards educational status, 228 participants (37.3%) were in grades 9–12.

Regarding military characteristics, most respondents were non-commissioned officers [507 (82.6%)], mean age at first deployment was 25 years with a standard deviation of ±3.1, and the majority of the respondents [526 (85.9%)] had more than one deployment (**Table 1**).

**Table 1:** Sociodemographic and military characteristics of military personnel, Northwest Command Level Three Military Hospital, Ethiopia, 2022 (*n* = 612).

Sociodemographic characteristics		Frequency	Percentage
Age	18–24	128	20.9
	25–29	206	33.7
	≥30	278	45.4
Sex	Male	466	69.3
	Female	146	30.7
Religion	Orthodox	261	42.6
	Muslim	162	26.5
	Protestant	189	30.9
Marital status	Married	186	30.4
	Single	386	63.1
	Divorced/Widowed	40	6.5
Educational status	Grades 1–8	174	28.4
	Grades 9–12	228	37.3
	Diploma and above	210	34.3
Military characteristics			
Service in military	<5	413	62.5
	≥5	199	37.5
Rank	Commissioned officer	103	17.2
	Non-commissioned officer	507	82.8
Branch	Army	524	85.6
	Airforce/special force/mechanized	88	14.4
Nature of duty	Combat	499	81.5
	Combat supporter/services supporter	113	18.5
Number of deployments	1–3 deployment	371	60.6
	4–5 deployment	122	19.9
	≥6 deployment	119	19.5
Length of deployment in months	1–5 months	258	42.2
	≥6 months	354	58.8
Age at first deployment	<25	290	47.4
	≥25	322	52.6
Time spent in forward area in months	1–2 months	118	19.3
	3–4 months	179	29.2
	5–6 months	153	25.0
	≥7 months	162	26.5

### Substance-related factors

**Figure 1:**
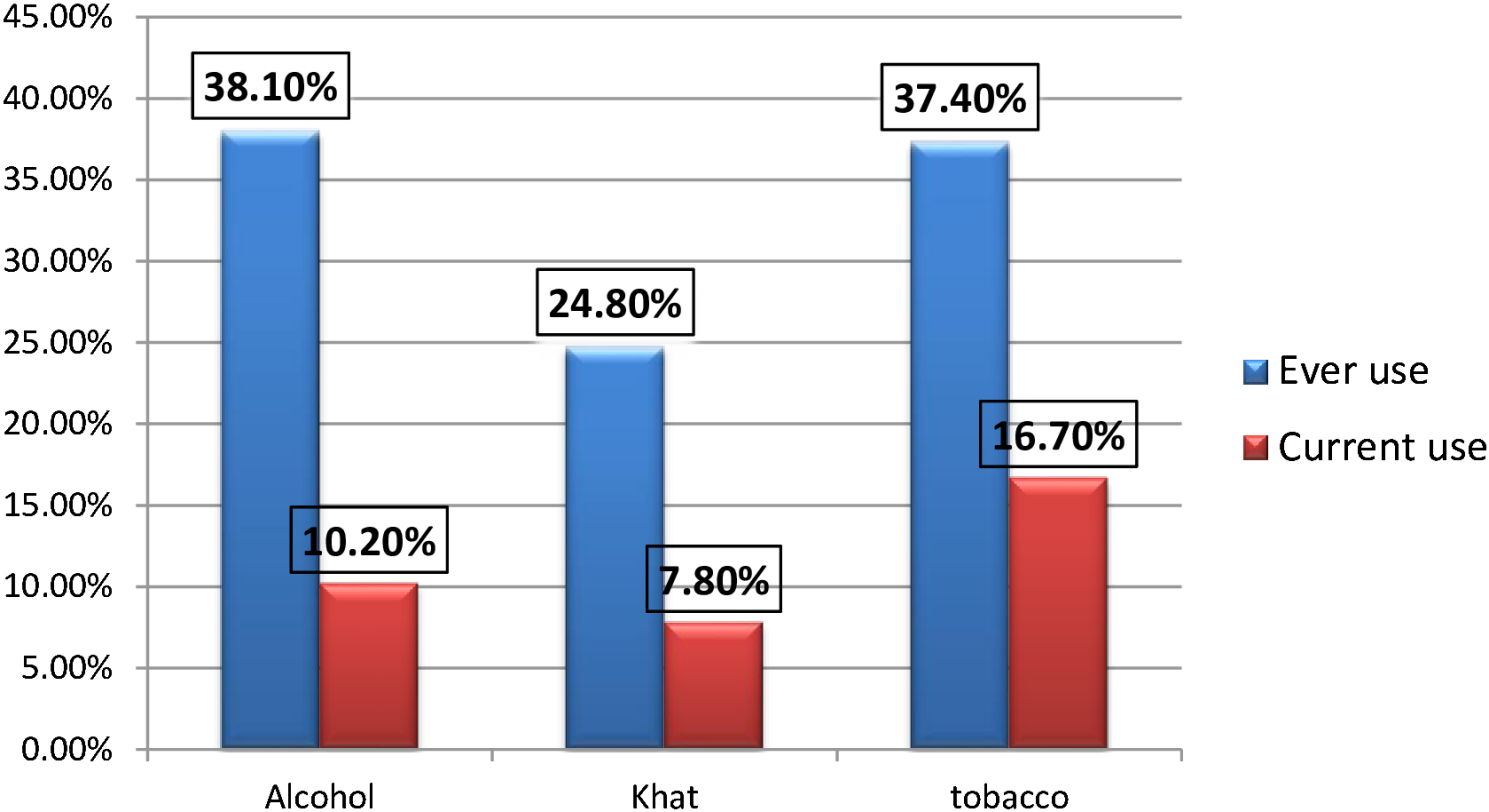
Showing the distribution of substance-related factors of the respondents among military personnel Northwest command level three military hospital, Ethiopia 2022 (n=612).

Out of the 612 participants, 234 individuals (38.1%) reported ever use of alcohol, while approximately 10.2% of the respondents indicated current alcohol consumption (**Figure 1**).

### Clinical factors of the respondents

Approximately 40 participants (6.5%) reported a family history of mental illness, while 62 participants (10.1%) had a known history of chronic medical illness. Overall, 172 (28.1%) of all participants were found to have depression (**Table 2**).

**Table 2:** Clinical factors of the respondents (military personnel, Northwest Command Level Three Military Hospital, Ethiopia, 2022; *n* = 612).

Clinical factors	Category	Frequency	Percentage
Have any known diagnosed chronic medical condition	Yes	62	10.1
	No	550	89.9
Known family history of mental illness	Yes	40	6.5
	No	572	93.5
Known history of mental illness in the past	Yes	33	5.4
	No	579	94.6
Having depression	Yes	172	28.1
	No	440	79.1

### Combat trauma exposure factors

The majority of the respondents, 466 (76.1%) and 497 (81.2%), have experience handling dead bodies and have faced being shot or receiving small arms fire, respectively. Regarding combat exposure, 175 (28.6%) of the participants had high combat exposure (**Table 3**).

**Table 3:** Distribution of combat trauma exposure factors of the respondents (military personnel, Northwest Command Level Three Military Hospital, Ethiopia, 2022; *n* = 612).

Characteristics	Frequency	Percentage	
Ever been attacked or ambushed	450	73.5	
Receiving incoming artillery, rocket, or mortar fire	389	63.6	
Being shot at or receiving small arms fire	497	81.2	
IED (improvised explosive device)/booby trap exploding near you	335	54.7	
Aid wounded/shouted	438	71.6	
Handling dead body	466	76.1	
Hearing of a friend or co-worker being injured or killed	473	73.3	
Seeing enemy forces wounded, killed or dead	450	73.5	
Combat exposure			
Low combat exposure	102	16.7	
Moderate combat exposure	170	27.8	
High combat exposure	175	28.6	
Very high combat exposure	165	27	

### Psychosocial factors

Out of the 612 participants, almost half, 285 (45.9%), reported having strong social support. Additionally, one-quarter of the participants, 155 (25.3%), reported a high perceived life threat, while 94 individuals (15.4%) disclosed experiencing childhood physical abuse and neglect in the past (**Table 4**).

**Table 4:** Distribution of psychosocial factors of the respondents (military personnel, Northwest Command Level Three Military Hospital, Ethiopia, 2022; *n* = 612).

Characteristics	Category	Frequency	Percentage
Social support	Poor	281	29.9
	Moderate	148	24.2
	Strong	183	45.9
Brief resilience coping scale	Low resilience	168	27.4
	Moderate resilience	127	20.8
	High resilience	317	51.8
Perceive life threat	Low perceived stress	232	37.9
	Moderate perceived stress	225	36.8
	High perceived stress	155	25.3
Child hood abuse or neglect	Yes	94	15.4
	No	518	84.6

### Prevalence of PTSD

The prevalence of PTSD in this population was 21.9% (95% CI: 18.6, 25.2).

### Factors associated with post-traumatic stress disorder

To determine the association of independent variables with PTSD, bivariate and multivariate binary logistic regression analyses were carried out.

On the bivariate analysis of PTSD to each explanatory variable: female sex, number of deployments, nature of duty in the military, single, history of mental illness, family history of mental illness, experiencing childhood physical trauma and neglect, depression, combat exposures, brief resilience scale, handling dead bodies, aiding wounded, being attacked or ambushed, receiving in coming artillery, being shot at or receiving incoming small arms fire, and seeing enemy forces wounded, killed, or dead were found to be significant at a *p*-value less than 0.25.

These factors were entered into multivariable binary logistic regression for further analysis. In multivariate analysis, female sex, a larger number of deployments, combat participants, poor social support, low resilience copers, handling dead bodies, very high combat exposures, and depression were significantly associated with PTSD at a *p*-value less than 0.05.

Being female was 2.3 times more likely to develop PTSD as compared with those male respondents (AOR = 2.3, 95% CI; 1.3, 3.87). The odds of developing PTSD was three times higher among respondents who participated as combat personnel as compared with those respondents who participated as combat supporters and combat service supporters (AOR = 2.75, 95% CI: 1.44, 6.36). Handling dead bodies increased the odds of PTSD by 2.5 times (AOR = 2.5, 95% CI, 1.24, 5.02), whereas the odds of PTSD were 2.94 times higher with 4–5 deployments (AOR = 2.94, 95% CI: 1.63, 5.32) and 3.4 times higher with ≥6 deployments (AOR = 3.4, 95% CI, 1.95, 6.17) as compared to 1–3 deployments (**Table 5**).

**Table 5:** Bivariable and multivariable independent factors of PTSD among respondents (military personnel, Northwest Command Level Three Military Hospital, Ethiopia, 2022; *n* = 612).

Variable	Category	PTSD		COR (95% CI)	AOR (95% CI)
		Yes	No		
Sex	Female	48	98	2.16 (1.42, 3.28)	2.3 (1.37, 3.84)**
	Male	86	380	1	1
Nature of duty	Combat	120	379	2.23 (1.23, 4.84)	3.03 (1.44, 6.36)**
	Combat supporter/service supporter	44	99	1	1
Marital status	Married	47	139	1	1
	Single	78	308	0.75 (0.49, 1.13)	0.77 (0.46, 1.29)
	Widowed/divorced	9	31	0.86 (0.38, 1.93)	0.69 (0.26, 1.9)
Depression	Yes	73	99	4.58 (3.05, 6.87)	2.8 (1.68, 4.67)***
	No	61	379	1	1
Brief resilience coping	Low resilience	62	106	3.36 (2.16, 5.22)	2.02 (1.16, 3.54)*
	Moderate	25	102	1.41 (0.82, 2.41)	1.42 (0.74, 2.73)
	High resilience	47	270	1	1
Social support	Poor	62	121	3.18 (2.02, 5.02)	2.46 (1.4, 4.35)**
	Intermediate social	33	115	1.78 (1.06, 2.98)	1.3 (0.69, 2.41)
	Strong	39	242	1	1
Combat exposures	Low combat	10	92	1	1
	Moderate combat	19	151	1.16 (0.52, 2.59)	1.26 (0.49, 3.25)
	High combat	38	137	2.55 (1.21, 5.38)	2.18 (0.89, 5.32)
	Very combat	67	98	6.29 (3.05, 12.9)	4.8 (2.04, 11.39)***
Number of deployment	1–3 deployments	45	326	1	1
	4–5 deployments	38	84	3.28 (2.0, 5.37)	2.95 (1.63, 5.3)***
	≥6 deployments	51	68	5.43 (3.37, 8.77)	3.47 (1.95, 6.18)***
Family history of mental illness	Yes	13	27	1.79 (0.89, 3.58)	1.38 (0.58, 3.28)
	No	121	451	1	1
Previous history of mental illness	Yes	13	20	2.46 (1.19, 5.09)	2.32 (0.89, 6.04)
	No	121	458	1	1
Childhood physical abuse and neglect	Yes	25	69	1.36 (0.82, 2.25)	1.54 (0.81, 2.93)
	No	109	409	1	1
Been attacked or ambushed	Yes	104	346	1.32 (0.84, 2.08)	1.27 (0.71, 2.27)
	No	30	132	1	1
Receiving artillery, rocket, or mortar fire	Yes	91	298	1.28 (0.85, 1.92)	1.08 (0.63, 1.87)
	No	43	180	1	1
Being shot at or receiving small arms fire	Yes	112	385	1.23 (0.74, 2.05)	0.91 (0.46, 1.78)
	No	22	93	01	1
IED/booby trap exploding near you	Yes	86	249	1.65 (1.11, 2.45)	2.32 (0.89, 6.04)
	No	48	229	1	1
Aid wounded/shouted	Yes	106	332	1.67 (1.05, 2.64)	1.22 (0.69, 2.13)
	No	28	146	1	1
Handling dead body	Yes	119	347	2.99 (1.69, 5.33)	2.5 (1.25, 5.0)*
	No	45	131	1	1
Witnessed a friend being injured or killed	Yes	109	364	1.37 (0.84, 2.21)	1.17 (0.62, 2.23)
	No	25	114	1	1
Seeing enemy wounded, killed, or dead	Yes	104	346	1.32 (0.84, 2.08)	1.11 (0.67, 1.98)
	No	30	132	1	1

***p < 0.001, **p < 0.01, *p < 0.05, statistically significant; 1 = reference; model goodness of fit (Hosmer and Lemeshow) = 0.913. IED, improvised explosive device.

Respondents with low resilience were twice as likely to develop PTSD as those with high resilience (AOR = 2.0, 95% CI: 1.16, 3.53), and those with low social support had 2.46 times higher odds of PTSD compared to individuals with strong social support (AOR = 2.46, 95% CI: 1.39, 4.35). Additionally, the likelihood of developing PTSD was 4.8 times greater for respondents who experienced very high combat exposure relative to those with low exposure (AOR = 4.81, 95% CI: 2.03, 11.93). The odds of developing PTSD were 2.80 times higher among those respondents who had depression than those who had no depression (AOR = 2.8, 95% CI: 1.68, 4.67).

## Discussion

The military community at large, as well as those who have experienced combat battle in the war, may be adversely affected by PTSD. The prevalence of PTSD in this study was 21.9% (95% CI: 18.6, 25.2). This finding was in line with the studies conducted among US military personnel (25.1%) (42), Nepal’s army combatants (21.9%) (35), UK combat-injured military personnel (18.5%) (43), US soldiers exposed to combat (19%) (44), and admitted veterans in Nigeria (22%) (45).

On the other hand, the outcome of this study indicated a PTSD prevalence of 21.9%, which was lower than those of earlier studies on South African military veterans (33%) (46)., military veterans in China’s Xinjiang region (29%) (20), soldiers with amputation of a limb or spinal injury in Sri Lanka (41.7%) (47), New Zealand military personnel (30%) (34), US military service members (47%) (48), and US War Veterans Health Care clinic patients (37.8%) (49).

The possible reason for the high prevalence among Sri Lankan military personnel is that the study focuses on those who have suffered spinal injuries and amputations; as a result, individuals who have undergone amputations have higher rates of anxiety and depression (50), which are associated with PTSD. Furthermore, the severity of the trauma predicts the onset of PTSD (51). Likewise, there is increased prevalence in the study done in Western countries, the US (48, 49), and New Zealand (34). It might be that Ethiopia has intimate family structures and a stronger extended family system than Western nations. According to the study, strong support networks (family and community) can enhance resilience and help to mitigate the psychological effects of severe battlefield injuries, including PTSD (52, 53).

Furthermore, the research carried out in South Africa employed a non-probability sampling method that utilized convenience and snowball sampling techniques. Similarly, it could be a sample size difference, an instrument difference (lower PCL-C cutoff of <30 in the New Zealand military), a lower cutoff point that increases the magnitude of PTSD, and possible sociocultural differences that contribute to the difference.

This study had a PTSD prevalence of 21.9%, which is higher than the 15.5% reported in an earlier study at an Ethiopian military hospital (31). The difference might be that the current study was undertaken during the conflict, and those who have deployment-related injuries and combat exposure could be a possible factor, because combat exposure and minor wounds and injuries increase the prevalence of PTSD (54, 55). Additionally, the lifetime prevalence of PTSD is two to three times higher in the injured than in the uninjured (42).

The result of this study is also higher than studies done on hospitalized US soldiers with combat injuries (4.2%) (55), Australian veteran peacekeepers (16.8%) (18), Canadian armed forces (7.6%) (19), war veterans in Kosovo (11.2%) (56), UK armed forces personnel (3.5%) (57), and Nepali army personnel and veterans (9%) (58). The low PTSD prevalence rates in the research done in Kosovo (56) and Canadian armed forces (59) could be due to the fact that the research involved non-admitted and non-injured military personnel, which would contribute to a lower chance of reporting PTSD (60). The delay in starting the study and the occurrence of trauma 15 years after the insurgency in Nepal’s military army (58) and 8 years after the war in Kosovo might contribute to the difference (56). Evidence showed that after 1 year of the occurrence of trauma, the prevalence of PTSD decreased by 50% (61). Additionally, the difference might also be due to a difference in the instruments (Harvard Trauma Questionnaire-40 HTQ, WHO’s Composite International Diagnostic Interview).

As regards the independent predictors of PTSD among admitted military personnel, female sex, low social support, a greater number of deployments, depression, low brief resilience, very high combat exposure, and handling dead bodies are significantly associated with PTSD.

In this study, being female was significantly associated with PTSD. This finding was supported in previous studies (62–65). The possible reason might be that women have experienced more sexual and gender harassment than men. This is evidenced by the data indicating that half of military women face some type of gender harassment each year (66), and have experienced military sexual trauma each year (67), suggesting that gender harassment and military sexual trauma are negatively associated with mental health outcomes, including PTSD (68).

Respondents who participated as combat personnel were 3.0 times more likely to develop PTSD than respondents who participated as combat supporters and combat service supporters. The possible reason could be that combat personnel could have been more susceptible to enemy attack, deployment, and combat exposure (23). Data revealed a threefold increase in PTSD symptoms or diagnoses among deployed military personnel who reported combat exposures than among deployed military personnel who did not report combat exposures (68). This result is in line with previous studies (69, 70).

Participants who had a higher cumulative length of deployment (i.e., ≥4 deployments) were more likely to develop PTSD as compared with respondents with a lower cumulative length of deployment. This was supported by previous studies (69, 71). This is implicated by multiple deployments, which are thought to be an underlying factor in the high prevalence of substance use disorders (72). Thus, substance abuse prevents the body from naturally resolving trauma-related discomfort, increases physiologic arousal, and exacerbates PTSD symptoms (73).

Likewise, military personnel who had poor social support were 2.46 times more likely to develop PTSD as compared with those who had strong social support. This was also affirmed by previous studies (31, 69, 74–76). Findings consistently demonstrate that people who receive greater social support are better able to handle crises. In contrast, those with less social support could find it more challenging to heal from trauma (77).

The odds of developing PTSD were two times higher among respondents who had low resilience than among those who had high resilience. This was supported by a previous study (76). It is evidenced by a high level of resilience, which is a protective factor against unfavorable consequences, such as PTSD. People with high levels of resilience are less likely to experience PTSD symptoms after a traumatic event (52). In addition, low emotional resilience and stress response have been linked to deficits in the catechol-O-methyltransferase Val158 Met polymorphism, i.e., an enzyme that metabolizes important nucleotides for PTSD, which is shown to affect the risk of developing PTSD (78).

Military personnel who had very high combat exposures were 4.8 times more likely to develop PTSD as compared with those who had experienced low combat exposures. This was also affirmed by previous studies (23, 48, 55, 69, 79). Moreover, the odds of developing PTSD were 2.5 times higher among respondents who had experienced handling dead bodies than those who had not. Other studies also found that military members who have encountered high combat situations are at an increased risk of developing PTSD (31). Military personnel who had depression were 2.8 times more likely to develop PTSD than respondents who had no depression. This finding was supported by the results of previous studies (48, 56).

Overall, the military population is characterized by a notable prevalence of PTSD, which exerts a paramount influence on society, families, and individuals. The substantial impact of depression and other co-occurring conditions should not be overlooked and requires due consideration. Especially for individuals who have actively participated in combat and/or multiple deployments and have endured injuries and hospitalization during military activities, there exists a pressing need for comprehensive and easily accessible therapy and monitoring to effectively address their PTSD.

### Limitations

The cross-sectional nature of the study design might not show temporal relationships between PTSD and its predictors. There may be recall bias in some tools especially for the duration of deployment and number of deployments. Moreover, important factors such as sleep pattern that cause PTSD have been missed, which could predict PTSD.

## Conclusion

According to this study, the prevalence of PTSD in Ethiopia is significant among hospitalized military personnel. One in five admitted military personnel had screened positive for PTSD. Additionally, it was also found that female sex, low social support, a greater number of deployments, depression, low brief resilience, very high combat exposure, and handling dead bodies are significantly associated with PTSD. Moreover, the abovementioned military experiences and sociodemographic and clinical factor profiles need to be considered during the design and implementation of psychosocial interventions for military personnel. A longitudinal study is recommended to gain a better understanding of the causal relationship between PTSD and its associated risk factors.

## Data Availability

The datasets presented in this study can be found in online repositories. The names of the repository/repositories and accession number(s) can be found in the article/Supplementary Material.
